# The roles of PARP-1 and XPD and their potential interplay in repairing bupivacaine-induced neuron oxidative DNA damage

**DOI:** 10.18632/aging.202390

**Published:** 2021-01-20

**Authors:** Wei Zhao, Zhongjie Liu, Jiaming Luo, Changqing Ma, Luying Lai, Zhengyuan Xia, Shiyuan Xu

**Affiliations:** 1 Department of Anesthesiology, ZhuJiang Hospital, Southern Medical University, Guangdong Province, China; 2 State Key Laboratory of Pharmaceutical Biotechnology and Department of Anesthesiology, University of Hong Kong, Hong Kong; 3 Department of Anesthesiology, Affiliated Hospital of Guangdong Medical University, Zhanjiang, China

**Keywords:** DNA damage repair, oxidative stress, neurotoxicity, bupivacaine

## Abstract

Bupivacaine has been widely used in clinical Anesthesia, but its neurotoxicity has been frequently reported, implicating cellular oxidative DNA damage as the major underlying mechanism. However, the mechanism underlying bupivacaine-induced oxidative DNA damage is unknown. We, thus, exposed SH-SY5Y cells to 1.5mM bupivacaine to induce neurotoxicity. Then, iTRAQ proteomic analysis was used to explore the repair of neuronal oxidative DNA damage. By analyzing the STRING version 11.0 database, the bioinformatics relationship between key repair enzymes was tracked. Subsequently, immunofluorescence co-localization and immunoprecipitation were used to investigate the interaction between key repair enzymes. The iTRAQ showed that Poly [ADP-ribose] polymerase 1 (PARP-1) from the base excision repair pathway participated closely in the repair of oxidative DNA damage induced by bupivacaine, and inhibition of PARP-1 expression significantly aggravated bupivacaine-induced DNA damage and apoptosis. Interestingly, this study showed that there were interactions and co-expression between PARP-1 and XPD (xeroderma pigmentosum D), another key protein of the nucleic acid excision repair pathway. After inhibiting XPD, PARP-1 expression was significantly reduced. However, simultaneous inhibition of both XPD and PARP-1 did not further increase DNA damage. It is concluded that PARP-1 may repair bupivacaine-induced oxidative DNA damage through XPD-mediated interactions.

## INTRODUCTION

Local anesthetics (LAs) such as bupivacaine are widely used for regional anesthesia (RA) and pain treatment. However, application of local anesthetics at high concentration or a long period of continuous local anesthetic stimulation can cause neurotoxicity [1, 2]. The consequence of LA neurotoxicity can be severe once it occurs although the incidence of neurotoxicity due to the application of clinical concentrations of LAs is rare [3]. Bupivacaine is a widely used LAs in clinics. A growing number of studies shows that bupivacaine may be neurotoxic even when used at clinically relevant concentrations [1, 4], while the underlying mechanism is unclear.

Reactive oxygen species (ROS) are produced during cellular metabolism and DNA damage have been implicated in the process of bupivacaine-induced neurotoxicity. Studies show that DNA damage induced by oxidative stress is a major threat to the genome stability of neurons [5]. Also, oxidative stress-induced neuronal cell DNA damage is a mechanism of bupivacaine neurotoxicity. Activation of different repair pathways and their interactions are required during repairing of oxidative stress-induced DNA damage [6], which include nucleotide excision repair (NER), base excision repair (BER), homologous recombination (HR), strand break (single-and double-stranded) repair, and inter-strand crosslink (ICL) repair pathways [7].

**Figure 1:**
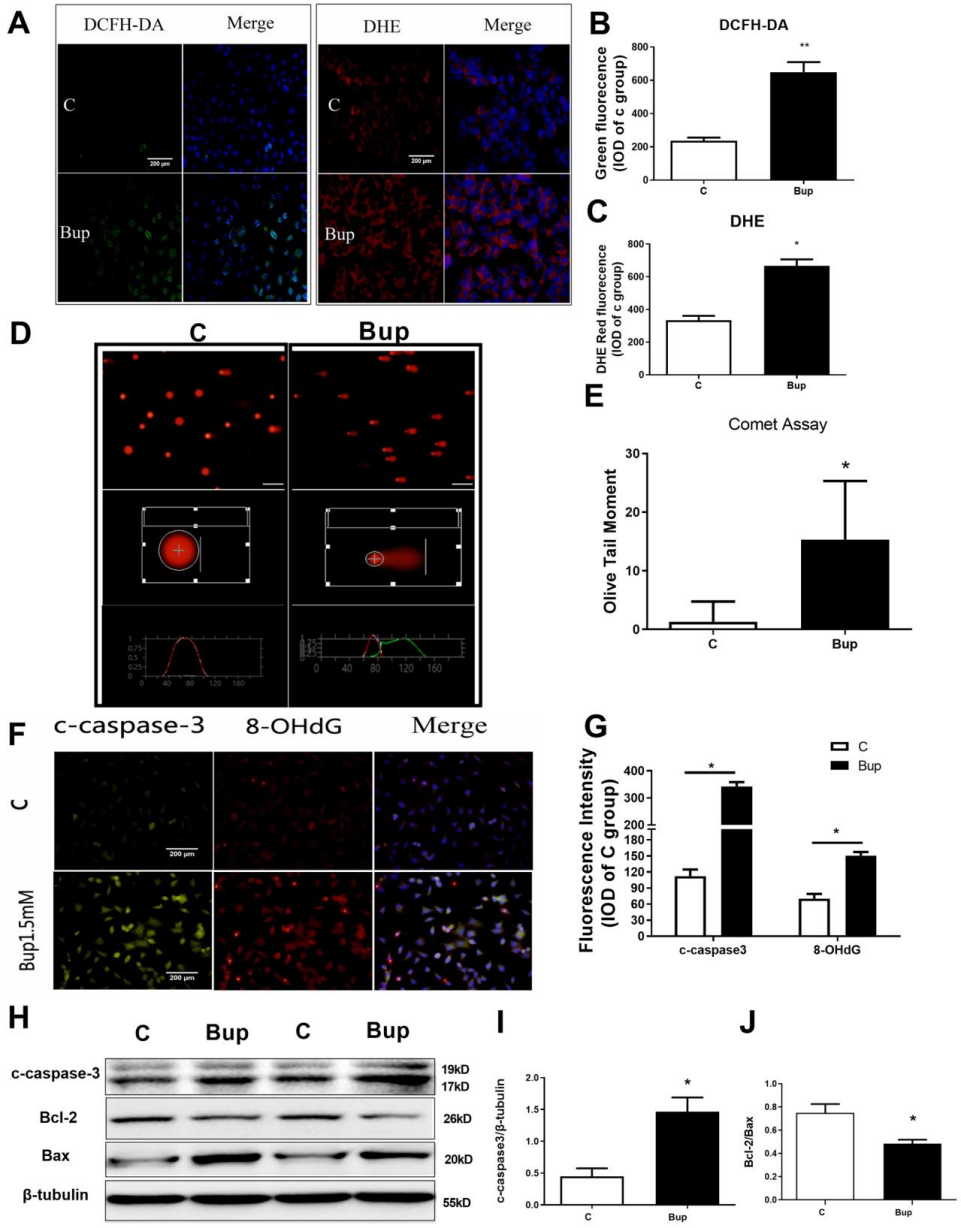
**Bupivacaine induced SH-SY5Y cell oxidative DNA damage and neurotoxicity.** After treating the SH-SY5Y cells with 1.5mM bupivacaine, the levels of intracellular reactive oxygen species (ROS) as stained by DCFH-DA (**A**, **B**) and superoxide anion as stained by DHE (**A**, **C**) were significantly increased. The index of DNA damage comet tail moment as assessed by in Comet Assay (**D**, **E**) were also robustly increased (* P <0.05). The oxidative DNA damage index 8-OHdG (**F**, **G**) increased. The expression of the apoptosis proteins cleaved-caspase3 (**F**, **G**, and **H**, **I**) increased while the apoptosis-related protein Bcl-2/Bax ratio (**H, J**) decreased. Data are the mean ± SD of three independent experiments, each performed in triplicates (*P <0.05, **P<0.01 vs control (**C**) group).

Recent evidence suggests that some proteins that primarily participated in NER also play a role in BER [8]. This suggests that any repair pathway will not exist alone but interact to repair different types of DNA damage [9]. It is unknown, however, which repair enzymes and pathways take part in the repair of neuronal oxidative DNA damage induced by bupivacaine, and whether there may exist interactions in between these repairing pathways. Our iTRAQ (isobaric tags for relative and absolute quantitation) proteomic screening on nerve cells with bupivacaine-induced neurotoxicity revealed that the expression of PARP-1, the key enzymes of the BER pathway, was significantly increased. The finding of the previous study [10] and Figure 1B of the current study both suggested that XPD (xeroderma pigmentosum D) also participated in the oxidative DNA damage of neurons caused by bupivacaine. Interestingly, we also found that there is an interaction between the two key repair enzymes instead of completing DNA repair alone. Further, our data demonstrate that PARP-1 may repair oxidative DNA damage through XPD-mediated interactions. It may provide a novel idea into the possible mechanisms and the subsequent development of preventive strategies against DNA damage caused by bupivacaine.

## RESULTS

### Bupivacaine induced oxidative DNA damage and neurotoxicity in SH-SY5Y cells

SH-SY5Y cell is an undifferentiated human neuroblastoma cell line [11]. Based on the results of our previous study [12], 1.5mM was selected as the concentration of bupivacaine for further research. After treated with 1.5mM bupivacaine, DCFH-DA (ROS) and DHE (superoxide anion) probes were used to detect the level of intracellular oxidative stress (Figure 1A–1C). Bupivacaine could significantly increase the level of oxidative stress. Comet assay was performed to test SH-SY5Y cell DNA damage, and bupivacaine (Bup) significantly increased Olive Tail moment (Figure 1D, 1E), an index of DNA damage. The expression of apoptosis index, cleaved-caspase3, and the oxidative DNA damage index 8-OHdG (Figure 1F, 1G) were tested by immunofluorescence (IF). We also found that the oxidative DNA damage index 8-ohdG (Figure 1F, 1G) was increased. Meanwhile, the expression of the apoptosis proteins cleaved-caspase3 (Figure 1F, 1G) increased. Then, Western blot was used to assess the expression of the apoptosis-related proteins cleaved-caspase3 (Figure 1H, 1I), Bax, and Bcl-2 (Figure 1H, 1J), and Bup significantly increased cleaved-caspase3 and reduced the ratio of Bcl2/Bax (Figure 1H–1J). These data showed that bupivacaine could induce SH-SY5Y cells oxidative DNA damage and apoptosis.

### iTRAQ proteomics screen identified the DNA repair pathways *in vitro*

**Figure 2:**
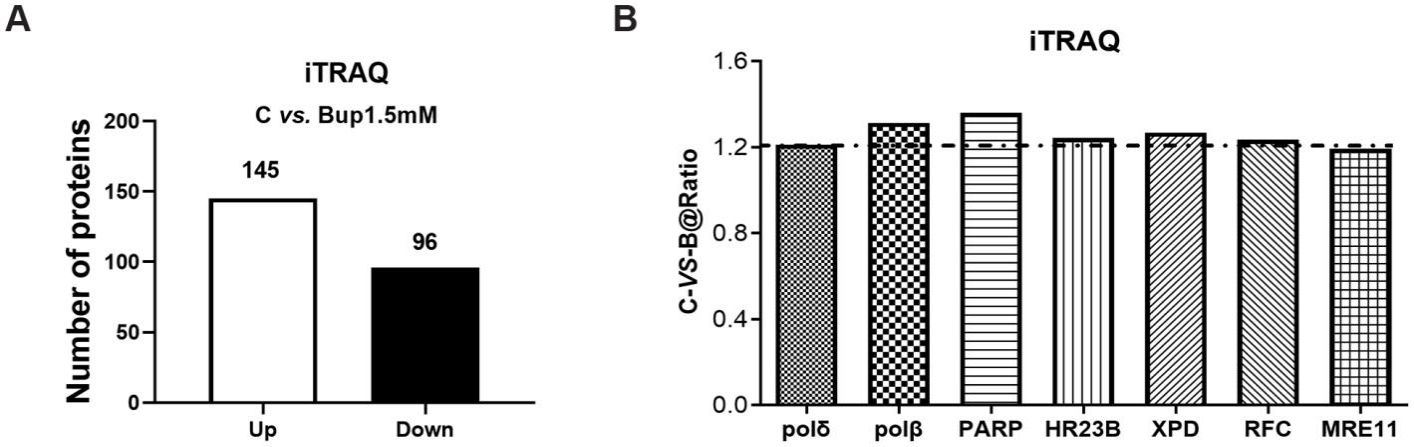
**The DNA damage repair proteins expression of SH-SY5Y cells after exposure to bupivacaine were detected by iTRAQ proteomic screening.** As showed in graph (**A**) iTRAQ proteomic screening results showed that: Of the total identified proteins, 241 proteins are significantly different between the (1.5 mM) bupivacaine and Control (C) groups, which included 145 upregulated and 96 downregulated proteins. Graph (**B**) displayed the list of DNA repair proteins whose expressions were increased by 1.2-fold or more after bupivacaine treatment (i.e., a ratio of Bup-vs-C greater than 1.2).

Accumulating evidence suggests that multiple DNA repair pathways participated in oxidative DNA damage repairing, including NER and BER [13, 14]. To investigate the key enzymes that may be associated with DNA repair pathways, iTRAQ proteomic was performed to test the expression of DNA repair proteins in SH-SY5Y cells after bupivacaine exposure. iTRAQ proteomic screening results showed that, 241 proteins were differentially expressed between cells treated with 1.5 mM bupivacaine (Bup) and the control (C) group, which included 96 downregulated and 145 upregulated proteins (Figure 1A). A list of all identified differentially expressed proteins was shown in Supplementary Table 1. Figure 2B displayed the repair proteins list whose ratio of Bup-*vs*-C is greater than 1.2. The differentially expressed repair proteins which enriched the base excision repair pathway were highlighted in red as shown in Supplementary Figure 1. Compared with the control group (Table 1), DNA repair enzymes activated by bupivacaine were mostly those in the BER pathway (PARP, polδ, ploβ) and NER pathways (XPD, HR23B, RFC, polδ). This finding is consistent with previous reports [15, 16].

**Table 1:** These differentially expressed repair genes and proteins are mainly those that are participated in the repair pathways: 1. base excision repair (BER); 2. nucleotide excision repair (NER).

**iTRAQ C-vs.-B (significant proteins)**	**Pathways names**	**KEGG NO.**
Polδ, ploβ, PARP	Base excision repair	map03410
HR23B, RFC, polδ, XPD	Nucleotide excision repair	map03420
polδ	Mismatch repair	map03430
MRE11	Non-homologous end-joining	map03450
MRE11	Homologous recombination	map03440

Pathways Names are the DNA damage repair pathways, KEGG NO. is the number of KEGG PATHWAY Database.

### The key DNA repair protein PARP-1 participated in the repair of neuronal oxidative DNA damage induced by bupivacaine

**Figure 3:**
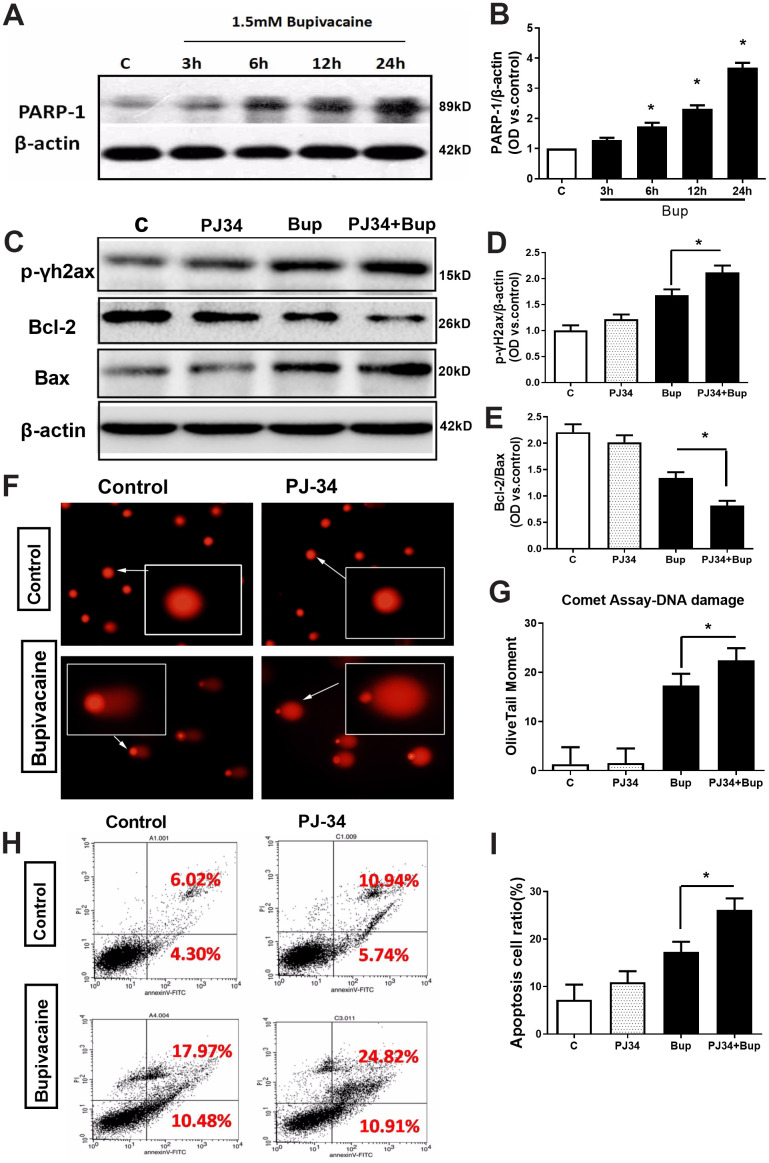
**The key DNA repair protein PARP-1 closely participated in the repair of oxidative DNA damage in neurons caused by bupivacaine.** In *in vitro*, the expressions of key repair protein PARP-1 in the BER pathway were significantly increased following bupivacaine-induced neuronal oxidative DNA damage. And, inhibition of PARP-1 expression with PJ34(a specific inhibitor of PARP) significantly aggravated the bupivacaine neurotoxicity. After SH-SY5Y cells were exposed to 1.5mM bupivacaine, the protein expression of PARP-1 (**A**, **B**) was increased obviously in a time-dependent manner. In the meantime, the DNA damage was aggravated: The DNA damage marker - phosphorylation level of γ-H2AX was significantly increased (**C**, **D**), while the comet assay indicator -the olive tail moment was significantly increased (**F**, **G**) in the Bupivacaine group as compared to Control group, which was concomitant with a significant reduction of the ratio of Bcl-2/Bax proteins (**C**, **E**) and increases of apoptosis as assessed by flow cytometry (**H**, **I**). Data are the mean ± SD of three independent experiments performed in triplicate, (*P <0.05, **P<0.01 vs C group).

The expression of key repair protein PARP-1 in the BER pathway was significantly increased after bupivacaine caused neuronal oxidative DNA damage. After SH-SY5Y cells exposure to bupivacaine, Western blot was used to detect the protein expression of PARP-1. The expression of PARP-1 (Figure 3A, 3B) increased significantly in a time-dependent manner. While inhibition of PARP-1 expression with PJ34 (a specific inhibitor of PARP) significantly aggravated the neurotoxicity. At the same time, the application of PJ34 to inhibit the repair protein PARP-1 further aggravated the DNA damage caused by bupivacaine, such that PJ34 and Bup combination could further increase p-γ-H2AX. The DNA damage marker phosphorylation level of γ-H2AX was significantly increased (Figure 3C, 3D), while the comet assay indicator -the olive tail moment was significantly higher (Figure 3F, 3G) in cells treated with both PJ34 and Bup. What is more, the apoptosis was also significantly increased which was manifested by the expression of apoptosis-related protein Bcl-2/Bax (Figure 3C, 3E) and the apoptotic cell death as assessed by flow cytometry (Figure 3H, 3I). Those data showed that the PARP-1 which is the key protein of the BER pathway closely participates in the repairing of bupivacaine induced neuronal oxidative DNA damage.

The XPD protein is part of the TFIIH complex that plays a role in both transcriptions and NER. Our previous study [16] and Figure 1B of the current study both suggested that XPD also participated in the oxidative DNA damage of neurons caused by bupivacaine. Therefore, it is reasonable to assume that the key repair enzyme-XPD of the NER pathway and the PARP-1 of the BER pathway both participated in repairing the oxidative DNA damage induced by bupivacaine. But the interaction between PARP-1 and XPD is still unclear.

### Possible existence of a novel interaction between XPD and PARP-1 in repairing the oxidative DNA damage caused by bupivacaine

**Figure 4:**
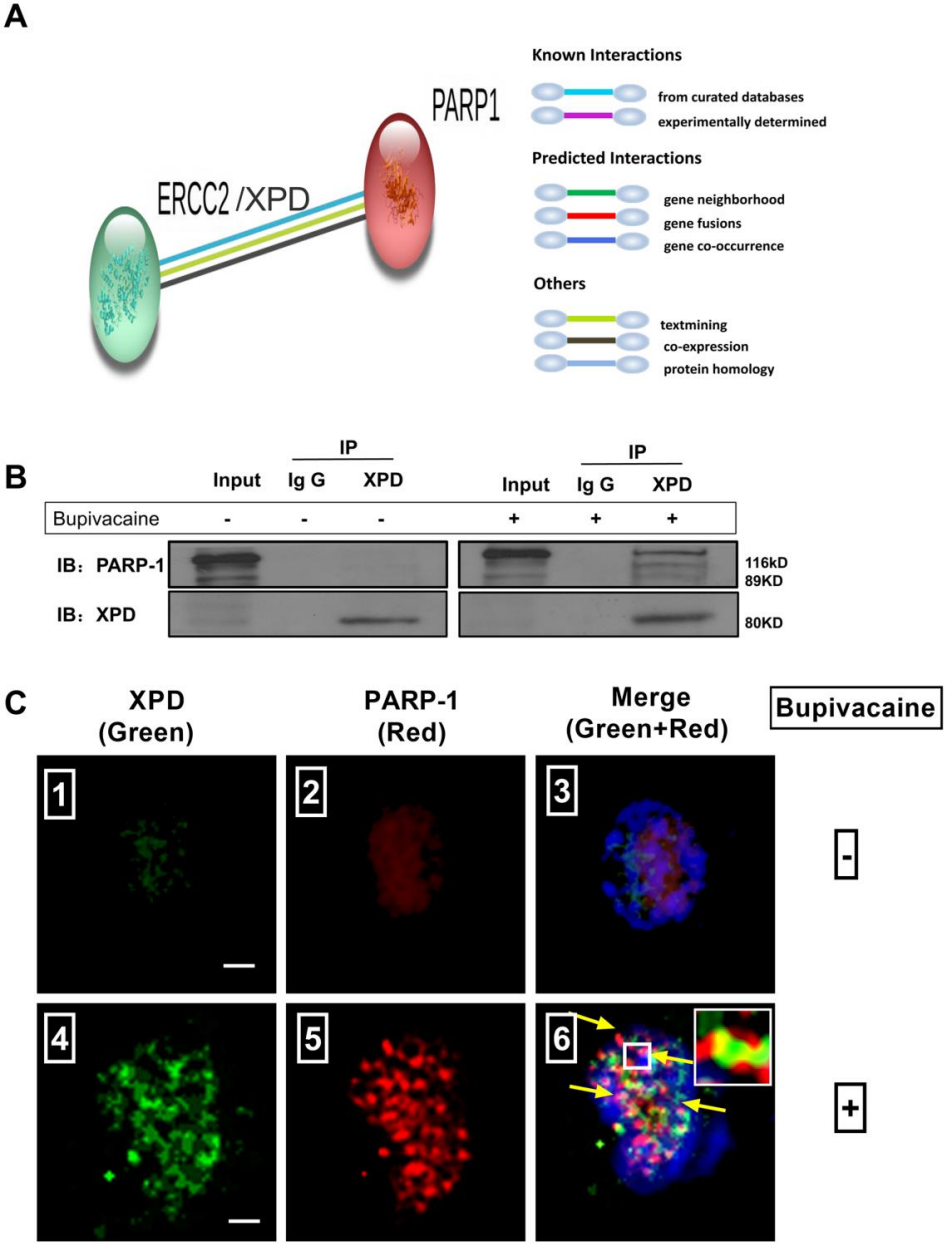
**Possible existence of a novel interaction between XPD and PARP-1 in repairing the oxidative DNA damage caused by bupivacaine.** Our previous study and iTRAQ results of the current study both suggested that XPD/ERCC2 also participated in the oxidative DNA damage of neurons caused by bupivacaine. But the interaction between PARP-1 and XPD is still unclear. Firstly, we identified the interaction between PARP-1 and XPD(ERCC2) using the STRING version 11.0 Program. (**A**) Combined screenshots came from the STRING website. Colored lines between the proteins indicate the various types of interaction evidence (according to the STRING website for color legend). Protein nodes that are enlarged indicate the availability of 3D protein structure information. No direct evidence or experimental data is available to confirm whether there exists an interaction between PARP-1 and ERCC2/XPD. For further verification, immunoprecipitation was applied to SH-SY5Y cells exposed to bupivacaine, and a strong interaction between XPD and PARP-1 was observed (**B**). Further, Immunofluorescence staining showed the colocalization (**C** ⑥, the yellow arrows) of XPD (Green) and PARP-1 (Red) following bupivacaine treatment in SH-SY5Y cells. Nuclei were stained with DAPI (blue). Bars, 5μm. Representative results of three experiments are shown.

Results of iTRAQ proteomic screening on nerve cells showed that the expression of NER enzyme-XPD and BER enzyme-PARP1 were significantly increased after the cells were treated with bupivacaine (Table 1). However, it is unknown whether there may exist any interactions between them. We, thus, identified the interaction between PARP-1 and XPD(ERCC2) using the STRING version 11.0 Program [17]. The colored lines showed the various types of interaction evidence (according to the STRING website for color legend (Figure 4A). To date, no direct evidence or experimental data exists to confirm the existence of an interaction between PARP-1 and XPD, but our results showed that both two proteins play a key role in repairing the oxidative DNA damage caused by bupivacaine.

To assess the novel interaction between the XPD and PARP-1, immunoprecipitation was used to determine (Figure 4B). Total protein was used for immunoprecipitation with the anti-XPD antibody. Immunoprecipitated (IP) proteins (line3 from left) were tested by anti-PARP-1 and anti-XPD antibodies. The left blots showed the levels of the proteins in the extract (10% of the input, line1 from left) with IgG control (line 2 from left). As expected, we observed a strong interaction between XPD and PARP-1. Immunofluorescence staining (Figure 4C) showed the colocalization (the yellow arrows) of XPD (Green) and PARP-1 (Red) following bupivacaine treatment in SH-SY5Y cells. Nuclei were stained with DAPI (blue). The results showed that XPD and PARP-1 can be co-localized, as showed in the orange focal point (Figure 4C).

### XPD via regulating PARP-1 mediated the repairing of bupivacaine-mediated oxidative DNA damage

**Figure 5:**
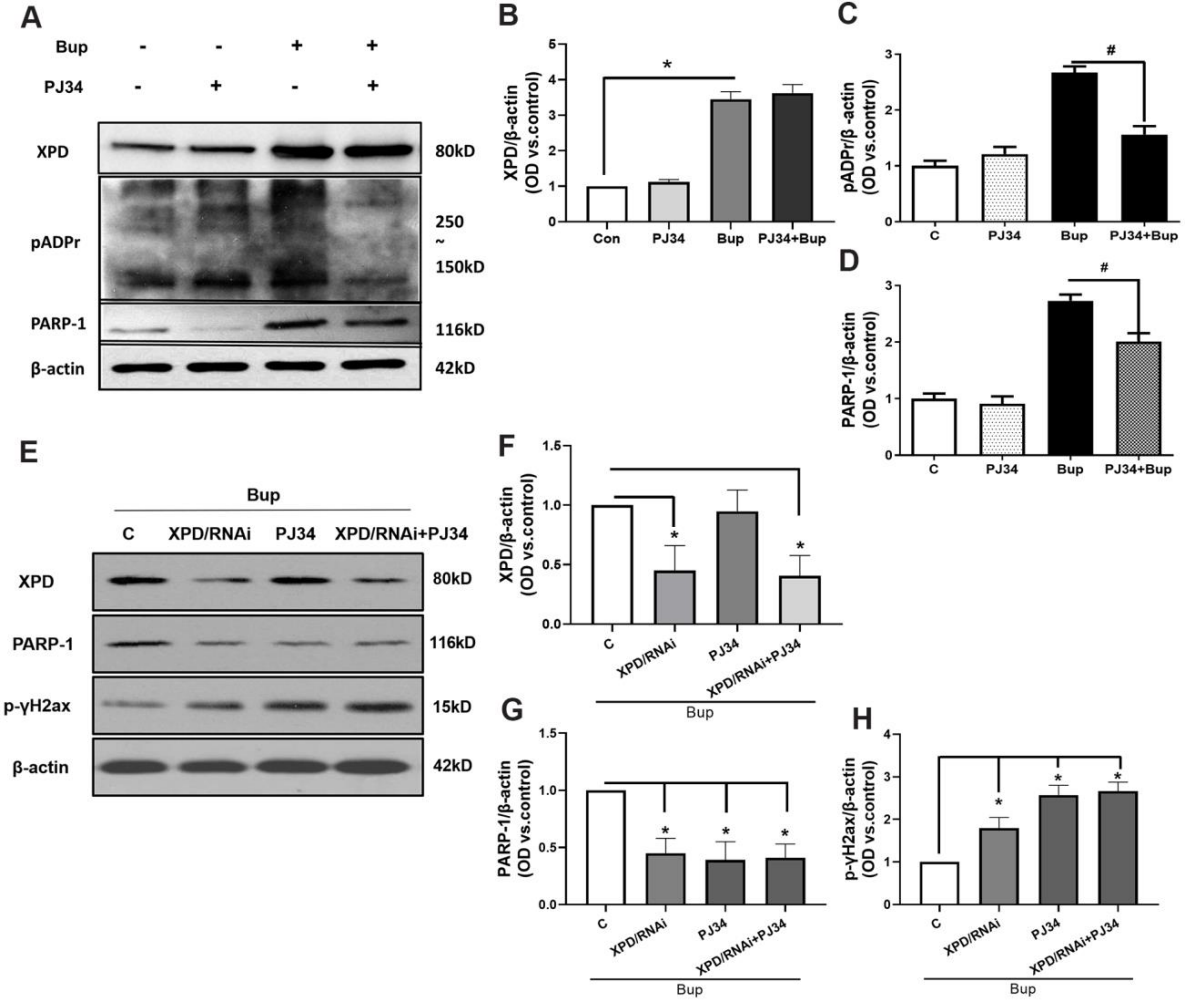
**PARP-1 via XPD-mediated interaction contributed to the repairing of bupivacaine-induced neuron oxidative DNA damage.** The protein expression of XPD and PARP-1 was examined in the presence or absence of PJ34(PARP-1 inhibition). Compared with the bupivacaine treatment alone group (Bup), the protein expression of pADPr (which represents the activation of PARP-1) was suppressed in cells treated with both PJ34 and Bup (PJ34+Bup group). After bupivacaine treatment, the expression of PARP-1 and XPD increased significantly. PJ34 can significantly reduce the expression and activity of PARP-1 caused by bupivacaine (**A**, **C**, **D**), but does not significantly inhibit the expression of XPD (**A**, **B**). Furthermore, the XPD-GV211-RNAi lentivirus was used to suppress the expression of XPD, while GV211-NC served as the control lentivirus group. PJ34 was used as the specific inhibitor of PARP-1. The expression of XPD was not affected after inhibition of PARP-1(**E**, **F**). However, after inhibiting the expression of XPD, PARP-1 expression was significantly reduced (**E**, **G**). Inhibition of either XPD or PARP-1 alone could increase the expression of the DNA damage index p-γ-H2AX induced by bupivacaine. However, concomitant inhibition of both XPD and PARP-1 did not further increase DNA damage (**E**, **H**). Data are the mean ± SD of three independent experiments performed in triplicate (*P <0.05, vs C group; ^#^P<0.05, vs Bup group).

The protein expression of XPD and PARP-1 was examined in the presence or absence of the PARP-1 inhibitor PJ34. Compared with the bupivacaine treatment alone group (Bup), the protein expression of pADPr (which represents the activation of PARP-1) was decreased after cell exposure to both PJ34 and Bup (PJ34+Bup group). After bupivacaine treatment, the expression of PARP-1 and XPD increased significantly. PJ34 can significantly reduce the expression and activity of PARP-1 caused by bupivacaine (Figure 5A, 5C, 5D), but does not significantly inhibit the expression of XPD (Figure 5A, 5B). Furthermore, the XPD-GV211-RNAi lentivirus was used to suppress the expression of XPD, while the GV211-NC served as the control lentivirus group. The expression of XPD was not affected after inhibition of PARP-1(Figure 5E, 5F). However, after inhibiting the expression of XPD, PARP-1 expression was significantly reduced (Figure 5E, 5G). Inhibition of either XPD or PARP-1 alone could increase the DNA damage index p-γ-H2AX expression induced by bupivacaine. But inhibiting both XPD and PARP-1 simultaneously did not further exacerbate DNA damage (Figure 5E, 5H). These data suggest that PARP-1 may be downstream of XPD, and both participate in the repairing of bupivacaine-induced neuronal oxidative DNA damage through the potential interaction.

## DISCUSSION

The neuroblastoma SH-SY5Y cell is a type of tumor cells with a low degree of differentiation from the human nervous system. This cell line is widely used in the mechanistic study for the pathogenesis and prevention and treatment of central nervous system diseases [18]. Recently, scholars have used it to study the mechanism of peripheral nerve cell toxicity of local Anesthetics [1]. In the *in vitro* experiments of local Anesthetic neurotoxicity, the concentrations used in the experiments were quite different. The common local Anesthetic, bupivacaine was widely used clinically for nerve block anesthesia. Cauda equina syndrome, a severe neurological complication, has been reported to be associated with bupivacaine spinal anesthesia when the concentration of bupivacaine applied was in the range of 0.5%-0.75% [19]. While the concentrations of bupivacaine were 1-1.5mmol/L [1] as used *in vitro* in the current study, it is equal to approximately 0.048% bupivacaine in the clinical setting. Moreover, the calculated IC50 value of bupivacaine was 1.2 mmol/L [1, 20]. Refer to our previous studies [12], in the SH-SY5Y cell line model, the IC50 (half maximal inhibitory concentration) of bupivacaine is about 1.5 mMol/L. So, this concentration of bupivacaine was selected as the drug concentration for the *in vitro* study in cells.

Oxidative stress is a state that breaks the oxidation/reduction homeostasis in the intracellular microenvironment. Our previous studies showed that bupivacaine can directly increase the intracellular oxidative stress of neurons [21, 22]. This may be an important potential mechanism for bupivacaine to cause neuronal toxicity. Evidence suggests that oxidative damage is the most common type of DNA damage [23]. Herein the results showed that bupivacaine can cause neuronal oxidative DNA damage *in vitro*. Oxidative stress/ROS induced cell DNA damage is mainly oxidative base damage. To deal with this type of oxidative damage, the repair is mainly accomplished through excision repair mechanisms such as BER and NER [24, 25]. Previous studies showed that bupivacaine can induce DNA damage in SH-SY5Y cells. The expression level of XPD, a repair enzyme in the excision repair pathway, was increased [16], and the expression of OGG1 in the base excision repair pathway was also significantly increased [15] in cells with local Anesthetic (e.g., bupivacaine) induced neurotoxicity. But, it is unclear which specific repair pathways and key repair proteins participate in repairing the oxidative DNA damage caused by bupivacaine. There may be tens or even hundreds of repair enzymes that are attributable to the repairing of bupivacaine induced DNA damage, which may represent a large and complex network of repair mechanisms.

iTRAQ technology is a method newly developed in 2004 by the American ABI company for isotope relative labeling and absolute quantification *in vitro* [26]. In this experiment, iTRAQ labeling technology was used to study proteomics of bupivacaine-induced neuronal DNA damage. First, the proteomes of the normal group and the injury model group were compared and quantified. The differentially expressed DNA damage repair-associated proteins were screened for high throughput. The above-mentioned assays, when combined with bioinformatics analysis, can lead to the conclusion that differentially expressed repair proteins are enriched in relevant DNA damage repair pathways. In the current experiment, we studied the DNA damage and repair mechanism of neurons caused by local anesthetic - bupivacaine. Due to the type of neuronal DNA damage caused by bupivacaine is also unclear, a specifically appropriate DNA damage agent cannot be found at the present. However, some studies have reported the use of iTRAQ experiments for common DNA damage stimulators that induce oxidative stress. Fan PC, et al [27] used a quantitative proteomic assay to study the mechanism of carbon ion irradiation on AHH-1 lymphoblastoid cells. An iTRAQ proteomic analysis was carried out to explore new cadmium which could generate DNA damage resistance determinants in the bacterium [28].

However, due to the limitations of the study, we cannot detect all the proteins of the DNA repair. It can only be studied based on the key repair proteins that may be participated in DNA damage repair reported in the relevant literature.

XPD is the most important restriction enzyme in NER which is the main pathway for DNA repair in mammalian cells [29]. It has been reported that PARP-1 [30] is a key enzyme in the process of BER and is a sensor of DNA damage [31]. Combined with the results of iTRAQ proteomics screening, both XPD and PARP-1 are the two common targets for screening. It is speculated that both of them play a key role in repairing neuronal DNA damage caused by bupivacaine. This is also an important reason for us to further verify the role of these two key repair enzymes XPD and PARP-1 in repairing neuronal oxidative DNA damage caused by bupivacaine.

One type of DNA damage can be repaired through a variety of different repair pathways. At the same time, a repair pathway can also participate in repairing different types of DNA damage. After iTRAQ proteomics screening, the bioinformatics analysis showed that the differential DNA repair genes in neurons after exposure to bupivacaine mainly participate in two repair pathways (i.e., NER and BER), which is similar to findings of previous studies [6]. However, in the previous studies, authors only addressed one of the repair pathways that may have participated in the repair of oxidative stress DNA damage. There is no clear evidence that two or more repair pathways may jointly participate in repairing the oxidative DNA damage.

The current study assessed the functional roles of PARP-1 in oxidative stress [32]. PARP-1 has been shown to target and modulate the DNA repair proteins at the sites of DNA lesions in the early steps [31]. These were the same result as demonstrated in our current study. Accumulating evidence shown that NER, besides BER, also participated in repairing oxidative DNA damage [24]. The XPD protein is part of the TFIIH complex that plays a role in both transcriptions and NER [33]. Here we propose that XPD participates in the repair of neuronal DNA damage induced by bupivacaine. After interfering with the expression of XPD, the DNA damage caused by bupivacaine was aggravated. This is similar to the research result of Lerner LK, et al [34]. However, this evidence cannot prove whether the treatment of bupivacaine affects the interaction between XPD and other subunits of TFIIH. It deserves further study. Our previous study and Figure 1B of this study both suggested that XPD also participated in the oxidative DNA damage of neurons caused by bupivacaine. Therefore, it is reasonable to assume that the key repair enzyme-XPD of the NER pathway and the PARP-1 of the BER pathway both participated in repairing the oxidative DNA damage induced by bupivacaine. But the interaction between PARP-1 and XPD is still unclear.

STRING version 11.0 program was used to find the interaction between PARP-1 with XPD(/ERCC2). From the STRING database, no direct evidence or experimental data is confirming the existence of an interaction between XPD and PARP-1, despite that there were reports in the past which suggested that PARP-1 may participate in multiple repair pathways [31, 35]. And there may exist a variety of different interactions with different DNA repair proteins during DNA damage repair. PARP-1, when being activated, forms the polymers of ADP ribose (pADPr or PAR) that post-translationally modify its target proteins. Xie et al. [36] have shown that recruitment of repair factors to DNA damage sites depends on the physical interaction with PARP-1, but is independent of PARP-1 activity. Mihaela Robu et al. [37] showed that the damaged DNA-binding protein 2(DDB2), a key lesion recognition protein of the global genomic sub-pathway of NER (GG-NER), associates with PARP-1 in the vicinity of UV-damaged chromatin. Furthermore, Keren et al. [38] described an association of E4orf4 with the DNA damage sensor PARP-1. E4orf4 reduces phosphorylation of the enzyme and inhibits its activity. PARP-1 inhibition assists E4orf4 in reducing adenovirus induced DDR signaling and improves the efficiency of virus replication.

**Figure 6:**
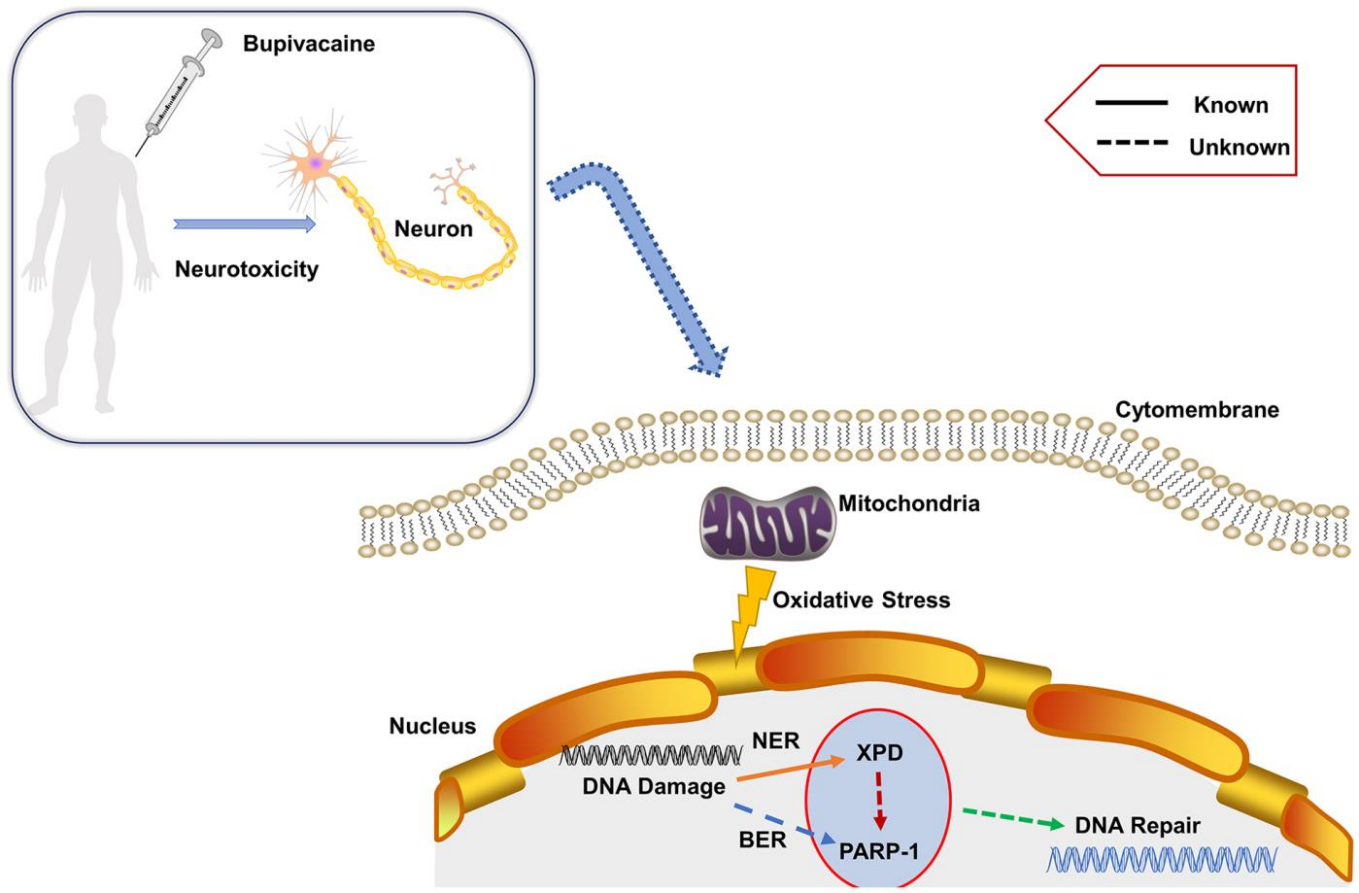
**Proposed mechanism.** Bupivacaine may cause oxidative DNA damage to neurons. The damaged DNA activates the largely unknown repair mechanism. Herein, our study showed that both PARP-1 and XPD closely participate in the oxidative DNA damage of neurons caused by bupivacaine. Interestingly, we also found that there is an interaction between the two key repair enzymes instead of completing DNA repair alone. Further, our data suggest that PARP-1 may repair oxidative DNA damage through XPD-mediated interactions.

Results from the current study firstly show that XPD and PARP-1 not only independently participated in repairing nerve DNA damage induced by bupivacaine, but there exist certain interactions. This suggests that in the process of repairing oxidative stress-induced DNA damage, there may be some cross-path between repair pathways that have not yet been discovered. Therefore, we propose the mechanism of this study as shown in Figure 6. The follow-up data suggest that PARP-1 may be downstream of XPD, and the interaction among them participated in repairing oxidative DNA damage of neurons caused by bupivacaine. But it is not clear whether XPD directly regulates or post-transcriptionally regulates PARP-1. It should be noted that XPD is a subunit of the transcription/DNA repair factor TFIIH, and is only functional when associated with the other subunit. Therefore, whether the treatment of bupivacaine affects the interaction between XPD and other subunits of TFIIH deserves further studies. While our current study demonstrated that XPD and PARP-1 interact in the repairing of bupivacaine-induced neuronal oxidative DNA damage, it remains to be answered whether XPD and PARP-1 interact directly or indirectly. Thus, it will be essential to detect the structural relation between XPD and PARP-1 in general and in the context of bupivacaine-induced neuronal injury repairing in specific in future studies.

The exact interaction between XPD and PARP-1 will provide new insights into the complex mechanisms of DNA damage repair pathways. It also may be a new target for further prevention of oxidative DNA damage in general and/or in bupivacaine-mediated oxidative DNA damage in specific.

## MATERIALS AND METHODS

### Chemicals and reagents

The Bupivacaine was purchased from Sigma-Aldrich, USA. DMEM/F12, fetal calf serum, and pancreatic enzyme (including or excluding) EDTA were purchased from Gibco, USA. PJ34 HCl was purchased from Selleck. Bupivacaine, 2,7-dichlorofluorescein diacetate (DCFH-DA), dihydroethidium (DHE) dye were purchased from Sigma-Aldrich, USA.

### Cell and culture

Neuroblastoma SH-SY5Y cell is a type of tumor cells with a low degree of differentiation from the human nervous system. The cytotoxic response of SH-SY5Y is similar to human primary neuronal cultures. Therefore, scholars have widely used it to study the mechanism of peripheral nerve cell toxicity of local anesthetics. Here, SH-SY5Y cells were purchased from the cell bank of the Chinese Academy of Sciences (Shanghai, China). The SH-SY5Y cells were maintained in DMEM/F12 medium supplemented with 10% FBS and 1% penicillin/streptomycin at 37° C in 5% CO_2_. The culture medium was replaced daily during cell growth. Cells were grown in 100-mm dishes and sub-cultured in 6-well (seeding density 5.0 × 10^5^ cells) or 12-well (seeding density 1.0 × 10^5^ cells) dishes. Experiments were conducted when cells reached 85% confluence. The SH-SY5Y cells were exposed to medium with 1.5mM bupivacaine for 3 h and then recovered in regular medium for 6h, 12h, 24h.

### Determination of reactive oxygen species (ROS) generation

Intracellular ROS generation caused by bupivacaine was tested by measuring the fluorescence intensity of DCFH-DA and DHE probe. After exposure to bupivacaine, SH-SY5Y cells were loaded with a final concentration of 10μM DCFH-DA or 5μM DHE dye at 37° C for 35 min. Non-fluorescent DCFH is converted to fluorescent DCF in proportion to the amount of generated intracellular ROS generation. Also, the SH-SY5Y cells were fixed in 4% paraformaldehyde at room temperature for 30 min, then washed with phosphate-buffered saline (PBS), stained with 1mg/ml DAPI for 5 min at room temperature in the dark. Finally, the samples were observed at excitation wavelength 504 and 524 nm (green fluorescence) emission or 488/610 nm (red fluorescence). DCFH-DA- and DHE-related fluorescence in SH-SY5Y cells was captured using a fluorescence microscope (Olympus, TH4-200, Japan) at 200x magnification. The fluorescence of images was calculated by Image-Pro Plus software in each of the five randomly selected fields.

### Comet assay

Single-cell gel electrophoresis assay (comet assay) [39] can measure DNA damage including single-strand breaks, double-strand breaks, alkali labile sites, and oxidative DNA base damage in single cells. The SH-SY5Y cells (2 × 10^5^ cells) were seeded in a 6-wells plate and the extent of DNA damage was measured by the related assay kit according to the instructions (Trevigen's Comet Assay® Kit). Cell images were obtained by a fluorescence microscope. At least, 50 randomly selected cells (from each of the two replicate slides) were analyzed with the Comet Assay Software Project (CASP-6.0, University of Wroclaw, Poland).

### Western blotting

After ultrasound treatments, cells were lysed in the lysis buffer by extracting proteins. After centrifugation, supernatant was taken as total protein. Bradford method was used for protein quantification. The same amount of protein extract was isolated by 10% SDS-PAGE and transferred to PVDF membranes (0.45μm, Millipore). The blots were blocked with 5% milk, then incubated overnight at 4° C with primary antibodies against p-γ-H2AX (Cell Signaling Technology, USA), cleaved-caspase3 (Cell Signaling Technology), Bax and Bcl-2 (Cell Signaling Technology), XPD (Cell Signaling Technology), PARP-1(Cell Signaling Technology), pADPr (Abcam, USA), β-actin (Cell Signaling Technology), β-tubulin (Cell Signaling Technology). Thereafter, these blots were incubated with HRP-conjugated secondary antibody, developed in ECL solution, and exposed onto hyper film (Amersham Biosciences, UK) for 1-10 min. The Image J software (NIH) was used to analyze the gray value of all bands.

### iTRAQ

The SH-SY5Y cells were disrupted in the lysis buffer (7 M Urea, 2 M Thiourea, 4℅ CHAPS, pH 8.5 40 mM Tris-HCl, 2mM EDTA, 1mM PMSF,) and sonicated in ice. Each group was performed in three biological replicates. Then samples were labeled with the 8-plex iTRAQ [26] reagent (Applied Biosystems) and Separated by High-performance liquid chromatography (HPLC) and analyzed by tandem Mass Spectrometry(MS) based on Q-EXECUTIVE. For protein quantification, a protein that contains at least two unique spectra was considered as had met the requirement for processing. The quantitative protein ratios were weighted and normalized by the median ratio in Mascot. We only used ratios with p-values < 0.05, and only fold changes of >1.2 or <0.8 were considered as significant. Identified proteins were classified according to annotations from the Uniport knowledge base (Swissport /TrEMBL, http://www.uniprot.org/) [10]. KEGG pathways present a set of molecules that participated in a biological system and an overview of their interactions in a sequence of coordinated events. The DNA repair pathway maps contain molecular interaction and reaction networks in which the differentially expressed genes participated. The differentially expressed genes are highlighted in red.

### Lentivirus mediated silencing of XPD

According to the XPD gene cDNA sequence, shRNA was designed and synthesized (Target Seq: TGGCCCTGATCATGGCATA), which was then annealed into the hU6-MCS-CMV-EGFP vector. After identified by sequencing, hU6-MCS- CMV-EGFP vector and packaging vector were co-transfected into SH-SY5Y cells. 72 hours later, the recombinant lentiviruses were obtained after harvesting and concentrating.

### PARP-1 activity inhibitor: PJ 34

PJ34 was purchased from Selleck Chemicals. The SH-SY5Y cells were treated with 20 nM PJ34 hydrochloride in phosphate buffer (vehicle) at 2 hours before treatment with Bupivacaine.

### Interaction analysis

The STRING database is used to collate information on all functional interactions between expressed proteins, by integrating known and predicted protein-protein association data for many organisms. STRING version 11.0 program was used to find the interaction between PARP-1 with XPD(/ERCC2). The STRING database is available online (http://string-db.org) [17].

### Immunoprecipitation

PARP-1 or XPD was immunoprecipitated from 500 to 1000 μg of total protein in RIPA lysis buffer as previously described with the following modifications. Primary antibodies (1:100 dilution) were incubated with total protein for 1 h at 4° C followed by the addition of protein A-agarose beads (Invitrogen - Fisher Scientific, USA) and further incubation for another 1 h at 4° C. The beads were isolated by centrifugation (1,000 g for 10 min at 4° C) and washed 3 times with RIPA buffer. Then, a 2×loading buffer was added to pellets and heated at 100° C for 10 min. All samples were resolved by SDS-PAGE gel as previously described.

### Immunofluorescence

In situ detection of XPD and PARP-1 was performed as previously described [12] with minor modifications. Briefly, XPD (1:50) and PARP-1 (1:100) antibodies were diluted in TBS buffer (1% fetal bovine serum, 0.05% Tween-20). Secondary antibodies were used at 1:500 (FITC-green) and 1:250 (cy3-red) to dilutions. Five images/groups were obtained using an LSM 510-META confocal with a 63 objective. For co-localization analysis, FITC (XPD) and cy3 (PARP-1) intensity measurements were obtained with individual masks for the respective channels, and co-localization was determined in Slide book 5.0 (Intelligent Imaging Innovations Inc., Denver, CO) using percent co-localization or Pearson’s correlation coefficient.

### Statistical analysis

In this study, no specific statistical methods were applied to predetermine sample sizes. However, our sample sizes are similar to previous studies. No data were excluded from the study. All the graphs and statistical data were generated using GraphPad Prism 5 (GraphPad Software, CA). Analyses were performed blind to genotype and experimental group. The unpaired t-test and one-way ANOVA (analysis of variance) followed by Tukey’s test were used for statistical analysis.

## Supplementary Material

Supplementary Figure 1

Supplementary Table 1
