# Supplementary material for: The roles of PARP-1 and XPD and their potential interplay in repairing bupivacaine-induced neuron oxidative DNA damage
Source: Aging (Albany NY). 2021 Jan 20;13(3):4274–90. doi: 10.18632/aging.202390 (PMC7906168; doi:10.18632/aging.202390)
Supplement: Supplementary Figure 1 [file aging-13-202390-s001.pdf]

SUPPLEMENTARY FIGURE

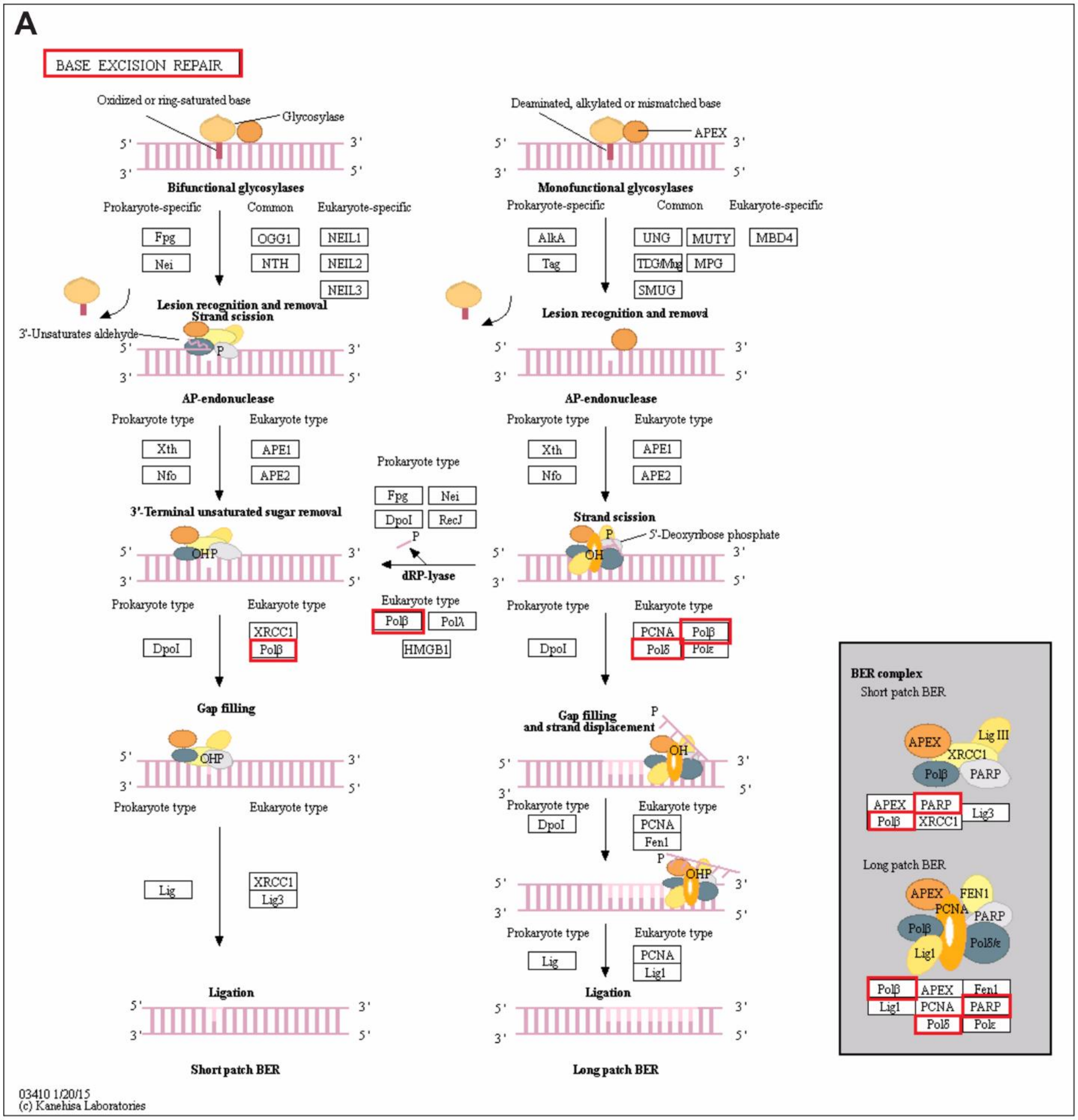

Supplementary Figure 1. The original picture in Figure 1A was from the KEEG database. The differentially expressed repair proteins which enriched the base excision repair pathway were highlighted in red as shown in Graph (**A**).
